# The upper respiratory tract microbiome of hospitalised patients with community-acquired pneumonia of unknown aetiology: a pilot study

**DOI:** 10.15172/pneu.2015.6/682

**Published:** 2015-12-01

**Authors:** Timothy L. Wiemken, Venkatakrishna Rao Jala, Robert R. Kelley, Paula Peyrani, William A. Mattingly, Forest W. Arnold, Patricio W. Cabral, Rodrigo Cavallazzi, Bodduluri Haribabu, Julio A. Ramirez

**Affiliations:** 111 Computational Epidemiology Unit, University of Louisville School of Medicine, Department of Medicine, Division of Infectious Diseases, Global Health Research Organization, 501 E. Broadway Suite 140B, Louisville, Kentucky 40202 USA; 211 Global Health Research Organization, University of Louisville, Louisville, Kentucky USA; 311 Department of Microbiology and Immunology, University of Louisville, Louisville, Kentucky USA; 411 Division of Pulmonary, Critical Care, and Sleep Disorders Medicine, University of Louisville, Louisville, Kentucky USA

**Keywords:** metagenomics, microecology, *Streptococcus pneumoniae*, bioinformatics, polymerase chain reaction

## Abstract

The composition of the upper respiratory tract microbiome may play an important role in the development of lower respiratory tract infections. Here, we characterised the microbiome of the nasopharynx and oropharynx of hospitalised patients with community-acquired pneumonia (CAP) with unknown aetiology in an attempt to obtain insight into the aetiology of CAP. A random sample of 10 patients hospitalised with CAP previously enrolled in a separate clinical trial (ClinicalTrials.gov registry, Study ID: NCT01248715) in which a complete microbiological workup was not able to define an aetiology were analysed in this pilot study. This larger trial (*n* = 1,221) enrolled patients from 9 adult hospitals in Louisville, Kentucky, USA. Nasopharyngeal and oropharyngeal swabs were obtained for metagenomic analysis. Polymerase chain reaction (PCR) for *Streptococcus pneumoniae* was performed in all patients. One patient had a distinct nasophararyngeal microbiome consisting largely of *Haemophilus influenzae*. This was the only patient with a negative PCR for *S. pneumoniae* in both nasophararyngeal and oropharyngeal specimens. Overall, substantial differences were found between nasophararyngeal and oropharyngeal microbiomes. The upper respiratory tract microbiome of only one patient suggested *H. influenzae* as a probable aetiology of CAP. Although this was a pilot study of only 10 patients, the presence of *S. pneumoniae* in the upper respiratory tract of the other 9 patients warrants further investigation.

## 1. Introduction

Community-acquired pneumonia (CAP) is the leading cause of infectious disease-related death in the United States [1]. The most common bacterial aetiology of CAP in hospitalised patients is *Streptococcus pneumoniae*, but other important pathogens include *Haemophilus influenzae, Staphylococcus aureus, Mycoplasma pneumoniae, Chlamydia pneumoniae*, and *Legionella pneumophila* [2]. One important clinical challenge in the management of hospitalised patients with CAP is that an aetiologic diagnosis cannot be reached in a significant number of patients even when applying state of the art microbiological methods [2]. Several possibilities may explain why an aetiology is not identified in some patients: 1) new unrecognised pathogens may be present; 2) current microbiological methods may not be sensitive enough to detect the pathogens; 3) some pathogens that we normally recognise as non-pathogenic colonisers of the upper respiratory tract may in fact be able to cause CAP.

Colonisation of the upper respiratory tract by pathogenic organisms, followed by microaspiration, is considered the primary pathogenesis of pneumonia [3]. It has been speculated that the normal flora of the upper respiratory tract may play an important role in the colonisation with pathogenic flora. However, this “normal flora” of the upper respiratory tract has not been well defined, particularly for patients with lower respiratory tract infections. With the new capabilities to fully characterise the human microbiota, the possibility now exists to better understand the bacterial communities in the upper airway.

We recently completed a prospective, randomised clinical trial evaluating the role of empiric antiviral therapy in the management of hospitalised patients with lower respiratory tract infections due to influenza virus (ClinicalTrials.gov registry, Study ID: NCT01248715). As part of this trial, hospitalised patients with CAP had a comprehensive microbiological workup including sputum and blood cultures; urinary antigen tests for *S. pneumoniae* and *L. pneumophila*; oropharyngeal swabs for polymerase chain reaction (PCR) detection of *M. pneumoniae, L. pneumophila*, and *C. pneumoniae*; as well as a nasopharyngeal swab for PCR detection of 12 respiratory viruses. An aetiologic diagnosis was not reached in many patients with CAP in whom this complete microbiological workup was performed.

In an attempt to better characterise the upper respiratory tract microbiome in hospitalised patients with CAP with unknown aetiology, we selected a random sample of 10 patients with CAP from our clinical trial in whom an aetiology was not identified. We speculated that in patients with CAP of unknown aetiology, distinct patterns of the microbiota in the nasopharynx and oropharynx might be identified. Furthermore, these patterns may provide insight into the aetiology of CAP in patients without identification of organisms using current technologies. With this in mind, we performed this pilot study with the objective to characterise the microbiome of the nasopharynx and oropharynx of hospitalised patients with CAP with an unknown aetiology.

## 2. Methods

### 2.1 Study design, patients and setting

The pilot study is a retrospective analysis of data collected prospectively from hospitalised adult patients with acute lower respiratory tract infection who were enrolled in a randomised clinical trial (NCT01248715). Enrollment in the clinical trial occurred from October 2010 through April 2013 in 9 hospitals in Louisville, Kentucky, USA. Briefly, all hospitalised patients with acute lower respiratory tract infections who were admitted to any of the 9 adult hospitals were approached for consent and enrollment into the clinical trial. More details on the enrollment and overview of patients are described on https://doi.org/clinicaltrials.gov/ct2/show/NCT01248715, and on the study website, https://doi.org/retostudy.com. Local institutional review board approval was obtained for each study site. In the clinical trial, all specimens with the exception of the nasopharyngeal and oropharyngeal swabs were prospectively collected during the initial days of hospitalisation for clinical care.

In the pilot study, the nasopharyngeal and oropharyngeal swabs were also prospectively collected after obtaining written informed consent from each patient. These swabs were then stored in our biorepository. Clinical data were collected from all patients on a paper case report form by trained study coordinators and/or research associates in our group. Data were then entered into a secure, online data capture system. All data entered into the data capture system were subsequently reviewed by trained clinical research coordinators and/or clinical research associates for data quality issues including out of range values, incorrect data types, and missing values. In the event data inconsistencies were identified, queries were sent to the individuals collecting and/or entering the data for follow-up. Once all queries were resolved, the cases were accepted into the database for analysis. The study protocol and the data collection form for the clinical trial are available online at https://doi.org/retostudy.com.

To validate the CAP diagnosis, the physician principal investigator involved in the pilot study reviewed all cases in the database. We assessed demographic, clinical history, laboratory, radiography, microbiological, therapeutic, and outcome data for all patients. A full list of variables collected for the study can be found on the case report form located on the study website, https://doi.org/retostudy.com/documents/RETOS_CRF.pdf. We included for the current pilot study a random sample of 10 patients satisfying the criteria for CAP and in whom a full microbiological workup failed to identify an aetiology from the 1,221 patients included in the intent-to-treat sample of the clinical trial (NCT01248715). A random number generator was used to select the random sample of patients.

### 2.2 Study definitions

CAP was defined as the presence of a new pulmonary infiltrate on chest radiograph at the time of hospitalisation that was associated with at least 1 of the following 3 criteria: new or increased cough; an abnormal temperature (<35.6°C or >37°C); or leukocytosis, leukopenia, or left shift pattern on white blood cell count.

A full microbiological workup was defined as having had the following laboratory tests: 1) blood cultures, 2) respiratory cultures (sputum, tracheal aspirate, bronchoalveolar lavage), 3) urinary antigen for *S. pneumoniae*, 4) urinary antigen for *L. pneumophila* serogroup 1, 5) nasopharyngeal swab for detection of respiratory viruses (influenza A virus untypable, influenza A virus H3 subtype, influenza A virus H1 subtype, influenza B virus, parainfluenza virus type 1, parainfluenza virus type 2, parainfluenza virus type 3, human metapneumovirus, respiratory syncytial virus [RSV] A, RSV B, adenovirus, and rhinovirus/enterovirus) via the Luminex xTAG Respiratory Viral Panel (Luminex Corporation, USA), 6) oropharyngeal swab for in-house polymerase chain reaction (PCR) detection of atypical pathogens: *L. pneumophila, M. pneumoniae, and C. pneumoniae*, and 7) rapid test for influenza virus.

Time to clinical stability was defined as the day when the patient met the following criteria: 1) normal or improved symptoms of pneumonia (cough and shortness of breath), 2) lack of fever for at least 8 h (<37.8°C), 3) white blood cell count normal or improving (decrease of >10% from baseline), and 4) oral intake considered adequate by the treating physician.

Length of hospital stay was defined as the number of days between hospital admission and hospital discharge.

### 2.3 Sample collection and storage

On the day of hospital admission, a nasopharyngeal swab (Copan Flocked Swab: Copan Diagnostics, USA) and an oropharyngeal swab (Copan Flocked Swab: Copan Diagnostics) was collected from hospitalised patients with CAP enrolled in our clinical trial. These samples were placed in 3.0 ml of universal transport media (Quidel, USA), vortexed for 30 s and aliquoted into 3 × 1.0 ml microcentrifuge cryogenic tubes, and stored at −80°C for future microbiome determination.

### 2.4 PCR

#### 2.4.1 DNA isolation and microbiome sequencing

The total genomic DNA from each swab was isolated with the Maxwell® automated DNA isolation method using the Maxwell® 16 Blood DNA Purification Kit (Promega Corporation, USA). The 16S rRNA genes were amplified using 16S rRNA-specific primers (0.5 µM), 27f (AGAGTTTGATCCTGGCTCAG) and 534r (ATTACCGCGGCTGCTGG) with the FastStart Hifidelity PCR System (Roche Diagnostics Corporation, Switzerland). These primers were anchored with adapters and multiplex identifiers (MIDs) for 454 sequencing to distinguish various samples in a single 454 sequencing reaction. The PCR cycling conditions were 95°C for 5 min, followed by 30 cycles of 94°C for 30 s, 56°C for 30 s, and 72°C for 1 min and 30 s with a final extension period of 8 min at 72°C. Each PCR reaction was performed in triplicate. The PCR amplicon products were pooled and gel purified using QIAGEN gel purification columns (QIAGEN, The Netherlands). The amplicon was quantified using the Quant-iT™ PicoGreen® dsDNA assay kit (Invitrogen: Thermo Fisher Scientific Inc., USA). The pooled, purified amplicons were sequenced using the 454 GS FLX System (Roche Diagnostic Corporation) or the 454 GS Junior System (Roche Diagnostic Corporation) according to manufacturer’s protocols.

#### 2.4.2 Microbiota sequence analysis

The microbial analysis was performed using the QIIME pipeline (QIIME 1.5.0: https://doi.org/www.qiime.org), developed and maintained by Knight and colleagues [4]. Briefly, the quality sequences (200–650 base pair lengths) were de-multiplexed based on their barcodes. The 16S rRNA Operational Taxonomic Units (OTUs) were picked based on 97% sequence identity using UCLUST [5] against the GreenGenes 16S rRNA database (https://doi.org/greengenes.lbl.gov/cgi-bin/nph-citation.cgi) (gg_otus-12_10) [6]. The GreenGenes taxonomies were used to generate the relative abundance of taxa at different levels of phylogeny (phylum, order, class, family, genus, species).

#### 2.4.3 Streptococcus pneumoniae PCR setup

PCR was used on the nasopharyngeal and oropharyngeal swabs to detect *S. pneumoniae* since the metagenomic analysis did not extend to the species level. The nested PCR protocols were used to detect the presence of *S. pneumoniae* as described by Murdoch [7]. The Roche FastStart HiFidelity PCR System (Roche Diagnostic Corporation) was used for amplification. Briefly, the first round PCR was targeted to the pneumolysin gene to amplify a 348 base pair region using primers la (5′-ATTTCTGTAACAGCTACCAACGA-3′) and lb (5′-GAATTCCCTGTCTTTTCAAAGTC-3′). The nested PCR (2nd round PCR, inner PCR) was performed using primers lla* (5′-CCCACTCTTCTTGCGGTTGA-3′) and llb (5′-TGAGCCGTTATTTTTTCATACTG-3′) on 1 µl to obtain a 208 base pair inner PCR product. We used clinically identified *S. pneumoniae* as a positive control and empty/TE buffer (Tris [10 mM]-EDTA [1 mM] pH 7.4)/mock isolation samples were used as a negative control.

### 2.5 Ethics statement

This study was conducted under the provisions of the Declaration of Helsinki. The prospective randomised clinical trial was registered prior to the enrollment of the first patient in the ClinicalTrials.gov registry (Study ID: NCT01248715, https://doi.org/clinicaltrials.gov/ct2/show/NCT01248715). The randomised clinical trial and the pilot study were also approved by the University of Louisville Human Subjects Program Protection Office (Protocol number 10.0465) prior to approaching patients for enrollment or data collection. Written informed consent was obtained from each patient by trained study coordinators and/or research associates.

## 3. Results

Baseline patient characteristics as well as clinical outcomes can be found in Table 1. The average age was 63 years (standard deviation [SD] = 16 years), 70% were male, and 50% were admitted directly to the intensive care unit. The average number of days required to reach clinical stability was 3.3 days (SD = 2.6 days), the average length of hospital stay was 5.6 days (SD = 5.6 days), all patients survived to discharge, and 2 patients had died at one-year follow-up.

**Table 1:** Baseline patient characteristics and clinical outcomes of hospitalised patients with community-acquired pneumonia with unknown aetiology

Case number	Age (years)	Sex	Days with symptoms prior to admission	PSI risk class	Admission location	Days to clinical stability^a^	In-hospital outcome	Days in hospital	One-year outcome
1	46	Female	7	IV	ICU	1	Alive	2	Alive
2	69	Male	Unspecified	IV	Ward	4	Alive	6	Alive
3	35	Male	10	IV	ICU	8	Alive	20	Dead
4	60	Male	3	IV	Ward	2	Alive	2	Alive
5	80	Female	3	IV	Ward	2	Alive	2	Alive
6	67	Female	1	V	ICU	8	Alive	9	Alive
7	91	Male	1	I	Ward	2	Alive	5	Alive
8	62	Male	4	I	ICU	3	Alive	5	Dead
9	64	Male	10	IV	ICU	1	Alive	2	Alive
10	60	Male	8	IV	Ward	2	Alive	3	Alive

PSI, Pneumonia Severity Index; ICU, intensive care unit

^a^Time to clinical stability was defined as the day when the patient met the following criteria: 1) normal or improved symptoms of pneumonia (cough and shortness of breath), 2) lack of fever for at least 8h (<37.8°C), 3) white blood cell count normal or improving (decrease of >10% from baseline), and 4) oral intake considered adequate by the treating physician

### 3.1 Bacterial phyla and genera

**Figure 1:**
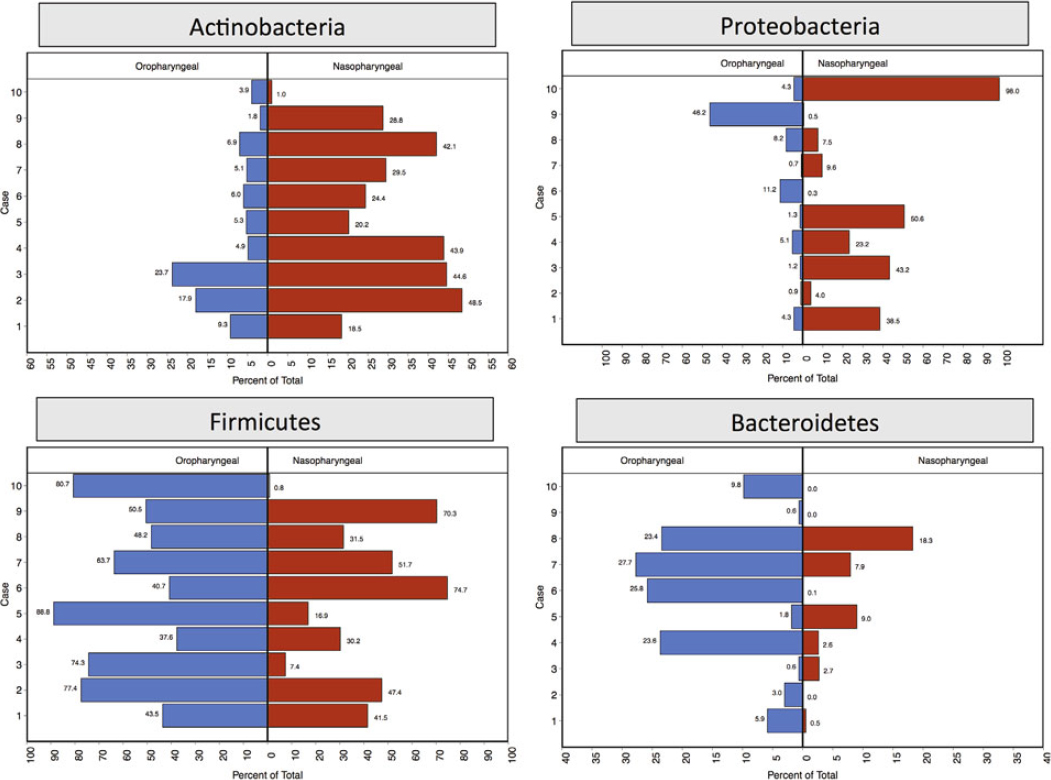
Proportions of bacterial phyla in the nasopharynx and oropharynx of hospitalised patients with community-acquired pneumonia with unknown aetiology

**Figure 2:**
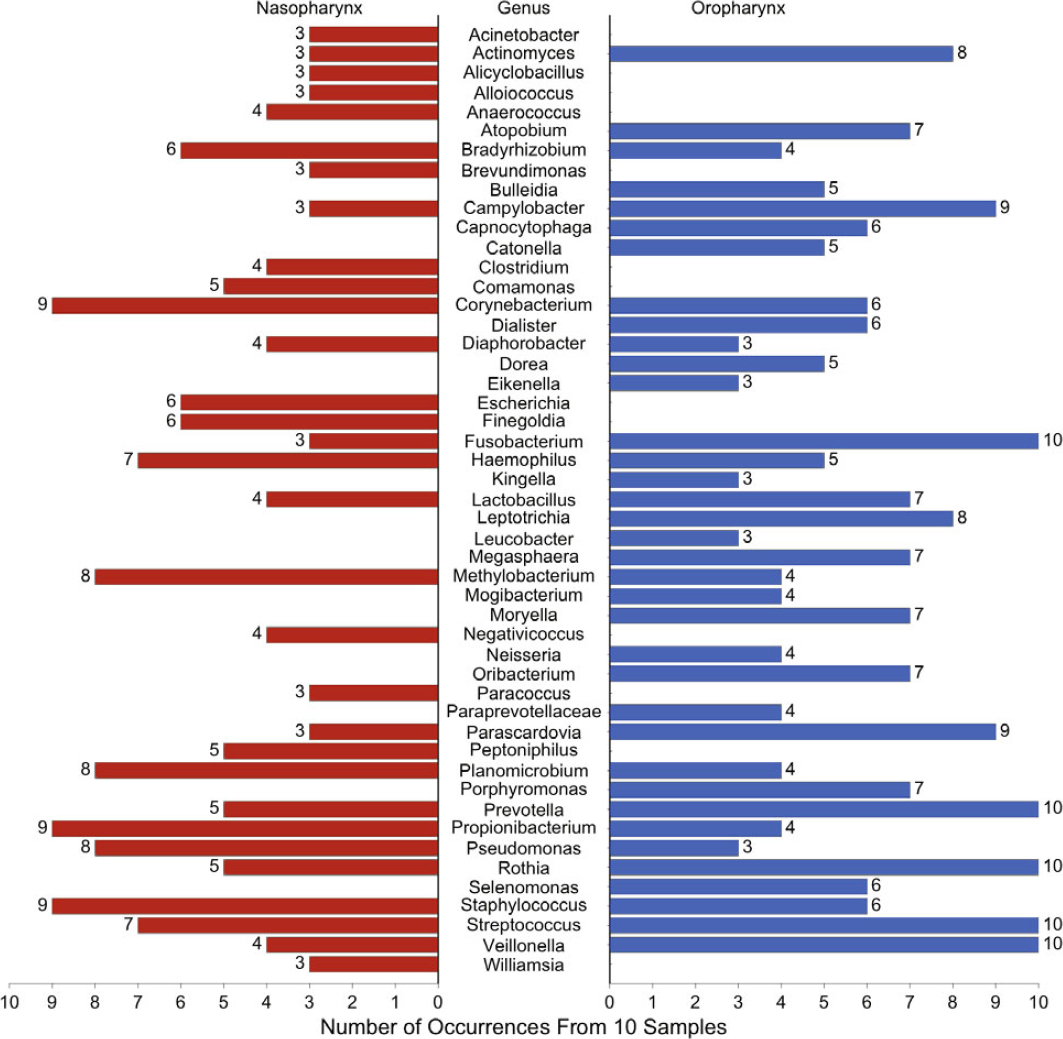
Proportions of bacterial genera in the nasopharynx and oropharynx of hospitalised patients with community-acquired pneumonia with unknown aetiology

For each patient, the relative percentages of the 4 most important phyla (Firmicutes, Bacteroidetes, Actinobacteria, and Proteobacteria) were plotted (Figure 1). There were clear phylum-level differences between the nasopharyngeal and oropharyngeal microbiota. Figure 2 depicts the most commonly identified genera in the nasopharynx and oropharynx.

### 3.2 Detection of Streptococcus pneumoniae by nested PCR

We only identified 1 sample (case number 10) that was negative for *S. pneumoniae* in both the nasopharyngeal and oropharyngeal swabs (Table 2). This sample had a high level of *H. influenzae* (98%) at the nasopharyngeal locaion.

**Table 2:** Nested PCR results for identification of *Streptococcus pneumoniae* in nasopharyngeal and oropharyngeal swabs

Case number	*S. pneumoniae* in nasopharyngeal swab	*S. pneumoniae* in oropharyngeal swab
1	+^a^	−^b^
2	+	+
3	−	+
4	+	+
5	−	+
6	+	−
7	+	+
8	+	−
9	+	−
10	−	−

^a^present

^b^absent

## 4. Discussion

We were able to identify 1 patient (case number 10) in whom the analysis of the upper respiratory tract microbiome provided some insight into the aetiology of CAP. This patient was unique with respect to the nasopharyngeal microbiome compared to other patients in our pilot study. The major difference was the substantially more Proteobacteria than Actinobacteria. Furthermore, the phyla Actinobacteria, Firmicutes, and Bacteroidetes were nearly absent in the microbiota in this patient’s nasopharynx. The primary organism within the Proteobacteria family was *H. influenzae*, which comprised 98% of the sequences. This was also the only patient with negative PCR for *S. pneumoniae* in both nasopharyngeal and oropharyngeal samples. These data suggest that *H. influenzae* may have been the aetiologic agent of this patient with CAP of unknown aetiology.

In our sample of patients, we found significant differences in the nasopharyngeal and oropharyngeal microbiomes. These data from patients with CAP is in accordance with recent literature suggesting that the upper respiratory tract microbiome of the nasopharynx is significantly different than the microbiome of the oropharynx in healthy humans [8]. Both microbiomes contain genera of organisms known to be aetiologic agents of CAP. This observation emphasises the need to consider the upper respiratory tract microbiome as two distinct microbial communities: the nasopharyngeal and the oropharyngeal microecosystems. Since microaspiration may originate from either of these two areas of the respiratory tract, future research in this area may need to involve both upper respiratory tract microecosystems.

The Human Microbiome Project indicates that the pharynx of normal individuals is inhabited by four primary phyla: Actinobacteria, Proteobacteria, Firmicutes, and Bacteroidetes. We also identified these four primary phyla in the nasopharynx and oropharynx of hospitalised patients with CAP. The most important pathogens causing CAP are present in the phyla Firmicutes and Proteobacteria. Firmicutes are Gram-positive bacteria that include the Streptococci such as *S. pneumoniae*, and the Staphylococci. The Proteobacteria include the Enterobacteriaceae, Pseudomonadaceae, and other Gram-negatives such as *Haemophilus* and *Legionella*. The Bacteroidetes are Gram-negative bacteria including anaerobes such as *Bacteroides*, capable of causing anaerobic lung infections. The Actinobacteria are Gram-positive bacteria that include several organisms able to cause sub-acute to chronic pneumonia such as *Actinomyces, Streptomyces*, and *Mycobacterium*. In our patients, we also identified several genera that included potential pathogenic organisms able to cause CAP.

Although the nasopharyngeal microbiome of hospitalised patients with pneumonia has not been well described, Chaban and colleagues [9] characterised the bacteria in this body site from patients with influenza infection. These investigators identified the nasopharyngeal microbiome to be relatively evenly distributed between Actinobacteria, Proteobacteria, and Firmicutes, with few Bacteroidetes. Our results showed a similar distribution for Actinobacteria and Firmicutes, but far less Proteobacteria overall. Lemon and colleagues [8] characterised the oropharyngeal microbiome of healthy adults and identified that it was composed largely of Firmicutes, with some cases having more Proteobacteria. This was much more closely aligned with our patients, with the largest phyla overall being Firmicutes.

The need to study both areas of the upper respiratory tract is underscored by our differential detection of *S. pneumoniae*. From the patients evaluated, the organism was identified in 90% of the patients’ nasopharynx and/or oropharynx. However, only 33% of these 9 patients had positive PCR in both the nasopharynx and oropharynx. It is unclear if the presence of this organism in the upper respiratory tract may indicate lower respiratory tract infection due to *S. pneumoniae*. Some investigators consider that *S. pneumoniae* is an under-identified aetiology of CAP due to poor sensitivity of current diagnostic techniques [3]. Since *S. pneumoniae* may only be colonising the upper respiratory tract of these patients, it is difficult to assess the potential for underdiagnosis of this pathogen as the cause of CAP in these patients. However, microaspiration from the upper respiratory tract plays an important role in the development of pneumonia due to *S. pneumoniae*. Furthermore, this pathogen is the most common cause of CAP. These two factors suggest that our documentation of *S. pneumoniae* in the upper respiratory tract of almost all of the patients with CAP of unknown aetiology may provide evidence that the aetiology of CAP in these patients may indeed be *S. pneumoniae*.

There are several limitations of our pilot study. First, since this was a pilot study, we evaluated only 10 patients, which limits the generalisability of our results. Patients signed the consent form and agreed to participate in our clinical trial on the day of hospital admission, but all patients had received antibiotics in the emergency department before enrollment into the trial. The prior antibiotic use before obtaining the nasopharyngeal and oropharyngeal samples may have biased our results. We were also not able to identify the microbiome down to species-level for all patients due to limitations in our methodology. Further, we looked at the microbiome only from the bacterial perspective. As the field of metagenomics evolves, the complex interactions between bacteria and viruses as aetiologies of CAP such as co-infection with *S. pneumoniae* and influenza viruses are becoming more relevant. In our study, we did not evaluate the upper respiratory tract microvirome, which may be important in patients with CAP. It is also possible that our high levels of detection of *S. pneumoniae* were due to detection of pneumolysin presence in some non-pneumococcal Streptococci inhabiting the upper respiratory tract of our patients.

Although our study is descriptive in nature, the data presented is the first step to begin to correlate the upper respiratory tract microbiome with the aetiology of CAP. Future studies of the microbiome in patients with identified aetiologies of lower respiratory tract infections may help to define the probable association of the upper respiratory tract microbiome with particular aetiologic agents of CAP. The upper respiratory tract microecosystems may predispose patients to colonisation with particular pathogenic organisms, influence virulence gene expression of pathogenic organisms already present in the normal microbiome, or impact the immune response to pathogenic organisms. If this were to be the case, reestablishing the upper respiratory tract microbiome homeostasis may have a role in protecting patients from lower respiratory tract infections.

In conclusion, this pilot study suggests that in hospitalised patients with CAP of unknown aetiology, characterising the upper respiratory tract microbiome may be of limited use in defining the aetiology of the patients’ lower respiratory tract infection. However, our results suggest that the nasopharyngeal and oropharyngeal microbiota of hospitalised patients with CAP of unknown aetiology are diverse, and contain several organisms that may be aetiologies of CAP. Furthermore, the presence of *S. pneumoniae* in the upper respiratory tract microbiome of these patients requires further investigation.
